# Advances of Molecular Markers and Their Application for Body Variables and Carcass Traits in Qinchuan Cattle

**DOI:** 10.3390/genes10090717

**Published:** 2019-09-17

**Authors:** Sayed Haidar Abbas Raza, Rajwali Khan, Sameh A. Abdelnour, Mohamed E. Abd El-Hack, Asmaa F. Khafaga, Ayman Taha, Husein Ohran, Chugang Mei, Nicola M. Schreurs, Linsen Zan

**Affiliations:** 1 College of Animal Science and Technology, Northwest A&F University, Yangling 712100, China; haiderraza110@nwafu.edu.cn (S.H.A.R.); rajwalikhan@nwafu.edu.cn (R.K.); mechugang@163.com (C.M.); 2 Department of Animal Production, Faculty of Agriculture, Zagazig University, Zagazig 44511, Egypt; samehtimor86@gmail.com; 3 Department of Poultry, Faculty of Agriculture, Zagazig University, Zagazig 44511, Egypt; dr.mohamed.e.abdalhaq@gmail.com; 4 Department of Pathology, Faculty of Veterinary Medicine, Alexandria University, Edfina 22758, Egypt; Asmaa.Khafaga@alexu.edu.eg; 5 Department of Animal Husbandry and Animal Wealth Development, Faculty of Veterinary Medicine, Alexandria University, Edfina 22578, Egypt; Ayman.Taha@alexu.edu.eg; 6 Department of Physiology, University of Sarajevo, Veterinary Faculty, Zmaja od Bosne Sarajevo 9071000, Bosnia and Herzegovina; husein.ohran@vfs.unsa.ba; 7 Animal Science, School of Agriculture and Environment, Massey University, Palmerston North 4442, New Zealand; N.M.Schreurs@massey.ac.nz; 8 National Beef Cattle Improvement Center, Northwest A&F University, Yangling 712100, China

**Keywords:** gene, markers, body, carcass, Qinchuan, cattle

## Abstract

This review considers the unique characteristics of Chinese cattle and intramuscular fat content (IMF) as factors influencing meat quality, including tenderness, flavor, and juiciness of meat. Due to its nutritional qualities, meat contributes to a healthy and balanced diet. The intramuscular fat content and eating quality of beef are influenced by many factors, which can generally be divided into on-farm and pre-slaughter factors (breed, sex of cattle, age at slaughter, housing system, diet, and pre-slaughter handling) and postmortem factors (post-slaughter processing, chilling temperature, and packaging). Meat quality traits can also be influenced by the individual genetic background of the animal. Worldwide, the function of genes and genetic polymorphisms that have potential effects on fattening of cattle and beef quality have been investigated. The use of DNA markers is recognized as a powerful and efficient approach to achieve genetic gain for desirable phenotypic characteristics, which is helpful for economic growth. The polymorphisms of the *SIRT4*, *SIRT6*, *SIRT7*, *CRTC3*, *ABHD5*, *KLF6*, *H-FABP*, and *ELOVL6* genes for body and growth characteristics of cattle, and also for beef quality, are considered with the aim of highlighting the significance of beef intramuscular fat content, and that growth, body, and meat quality characteristics are polygenically regulated.

## 1. Introduction

Beef is favored in both domestic and foreign markets due to its high protein content, low cholesterol, essential amino acids content, minerals, vitamins, and also greater digestibility and absorption which can reach 95%. Meat which is subjected to modification to either enhance taste, or improve preservation, is called processed meat [1]. Over the past few decades, for cattle to have sustainable, efficient genetic gain and long-lasting progress in traits of economic importance, the cattle selection programs have increasingly utilized molecular genetic technologies because genetic improvement by traditional breeding systems slow due to the generational lag associated with progeny testing. Therefore, the characterization of quantitative trait loci permits biomarker-aided assortment of the economically important traits and provides faster genetic gain when candidate genes are correlated with the traits of economic importance [2]. Qinchuan cattle (*Bos taurus*) are an indigenous Chinese breed of cattle that is widely used in the production of beef due to its physiological and qualitative characteristics. Despite this, the Qinchuan cattle have a slower growth rate and a less developed carcass compared with other commercial breeds [3], and so the improvement of growth and carcass characteristics through a focus on genetics is a key feature of Qinchuan cattle production. An increased standard of living in China has seen an increased demand for beef which cannot be met by domestic beef production. Therefore, improving the production and quality of beef is a priority. The use of DNA markers to improve the carcass and quality characteristics of cattle was identified as a useful and effective tool for genetic gain [2,4,5,6]. Quantitative traits of the body and carcass of cattle are defined by a number of genes [7,8,9]. Therefore, the screening of candidate genes is necessary to understand the connection between gene variation and body and carcass traits [10]. The *SIRT4*, *SIRT6*, *SIRT7*, *CRTC3*, *ABHD5*, *KLF6*, *H-FABP*, and *ELOVL6* genes are some candidate genes that have been identified as regulating meat and carcass traits through metabolic controls, fatty acid oxidation, fat deposition, and lipid synthesis. Especially for lipid metabolism, the *SIRT4* gene in Qinchuan cattle seems to be a major metabolic regulator that modulates fat deposition [11]. Sirtuins, or silent information regulator genes (*SIRT6*), are members of the class III nicotinamide adenine dinucleotide (NAD)-dependent deacetylase [12]. *SIRT6* controls several pathways in the cell, including apoptosis, energy homeostasis, functions of the mitochondria, and longevity. *SIRT6* in mammals is mainly located in the nucleus and participate in metabolism processes and DNA restoration [13]. It is proposed that *SIRT6* controls the development of the animal body and modulates lipid and glucose metabolism on a local and systemic level [14]. The *SIRT7* gene affects the traits related to growth and meat quality in Qinchuan cattle [15]. The *ABHD5* gene is a catalyst of adipose triglyceride lipase (ATGL), which has an important function in triglyceride metabolism. It has been reported that the *ABHD5* gene is related to improve carcass quality traits and can also be used as a biomarker for selection in Chinese cattle [16]. The gene known as Kruppel-like factor 6 (*KLF6*) participates in the control of cell division, separation, and muscle growth. Raza et al. [3] showed that the *KLF6* gene is associated with ultrasound carcass measures, as well as larger body and carcass measurements. Many studies report that the polymorphisms and expression levels of *H-FABP* are related to the fat deposition in cattle [11]. The gene *ELOVL6* encodes an enzyme that participates in lipogenesis via the catalytic elongation of both saturated and monounsaturated fatty acids. It has been shown that the *ELOVL6* gene regulates the lipid metabolism and adipocyte proliferation in Qinchuan cattle [17]. By controlling the gene expression in the cell-cycle, this gene stimulates adipocyte proliferation.

The current review was intended to identify the genetic polymorphisms of bovine genetic biomarkers that are associated with body and carcass traits in Qinchuan cattle. The identified SNPs in Qinchuan cattle can be used to improve our understanding about the genes overall to inform on their value for marker-assisted selection of body, carcass, and meat traits of Qinchuan cattle.

## 2. Effect of Some Slaughter Value Factors on Meat Quality

**Figure 1:**
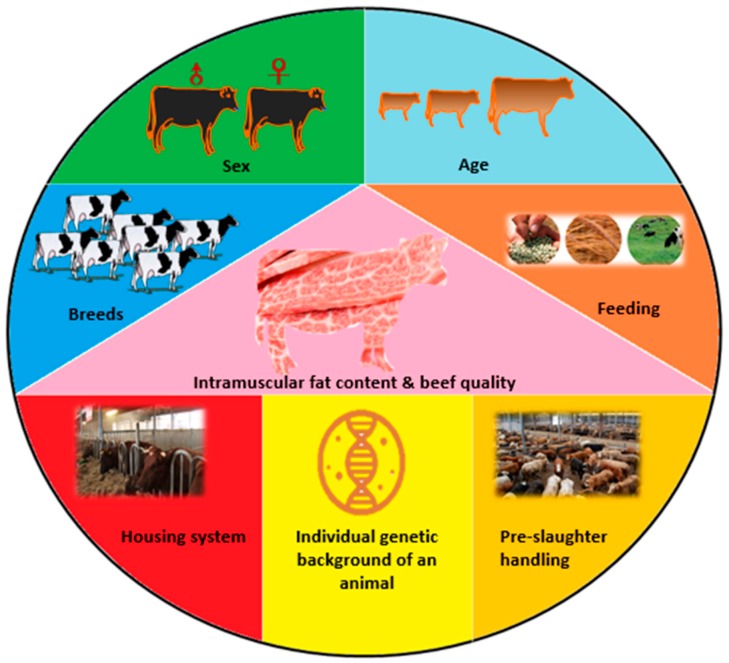
Drivers of meat quality.

Numerous studies have demonstrated that intramuscular fat content of cattle and beef quality are influenced by factors of the production or processing system including the breed, sex, age, housing system, feeding, and pre-slaughter handling, but also the individual genetic background of an animal [18,19] (Figure 1). The impact of breed on suitability for meat production was reported by many authors, but it is difficult to pinpoint the best breed for beef production and it is unlikely that one breed will have all the desirable traits for survival, growth, and quality meat production [20,21,22]. Breeds differ in many aspects including degree of muscling, intramuscular fat content, meat aroma, juiciness, and tenderness. Because cattle breeds originating from *Bos indicus* exhibit late maturation, higher calpastatin activity, and a larger percentage of connective tissue, the meat of these animals is characterized by lower tenderness and higher cooking loss compared to the meat of the breeds derived from *Bos taurus* [23].

## 3. Most Important Beef Quality Traits

Beef quality attributes of importance for consumers include color, tenderness, taste, and juiciness [24]. A significant role is also played by freshness, low fat content, and high nutritive value [25]. Color depends on the concentration and chemical form of myoglobin found in the muscle tissue. Fresh meat contains deoxymyoglobin (DMb), oxymyoglobin (OMb), and metmyoglobin (MMb). The DMb form gives a scarlet pigmentation to fresh meat. In the presence of oxygen, deoxymyoglobin is oxidized to OMb, resulting in a bright pink-red color. When the above forms are oxidized to MMb, the meat becomes brown, which is highly undesirable. The formation of metmyoglobin is favored by factors such as low pH, increased salt concentration, and UV light. The enzyme metmyoglobin reductase decreases the level of the undesirable form of myoglobin, thus stabilizing the color of meat.

Beef tenderness is the ease of breakdown during biting and chewing. Many methods are available for instrumental determination of the degree of meat tenderness. Physical methods measure the force needed to shear, penetrate, detach, grind, compress, or tear a meat sample. The indicator typically used to evaluate tenderness is the shear force value, which measures the force needed to cut the sample perpendicular to the orientation of the muscle fibers. However, the correlation between this force and sensory tenderness is low [26].

**Figure 2:**
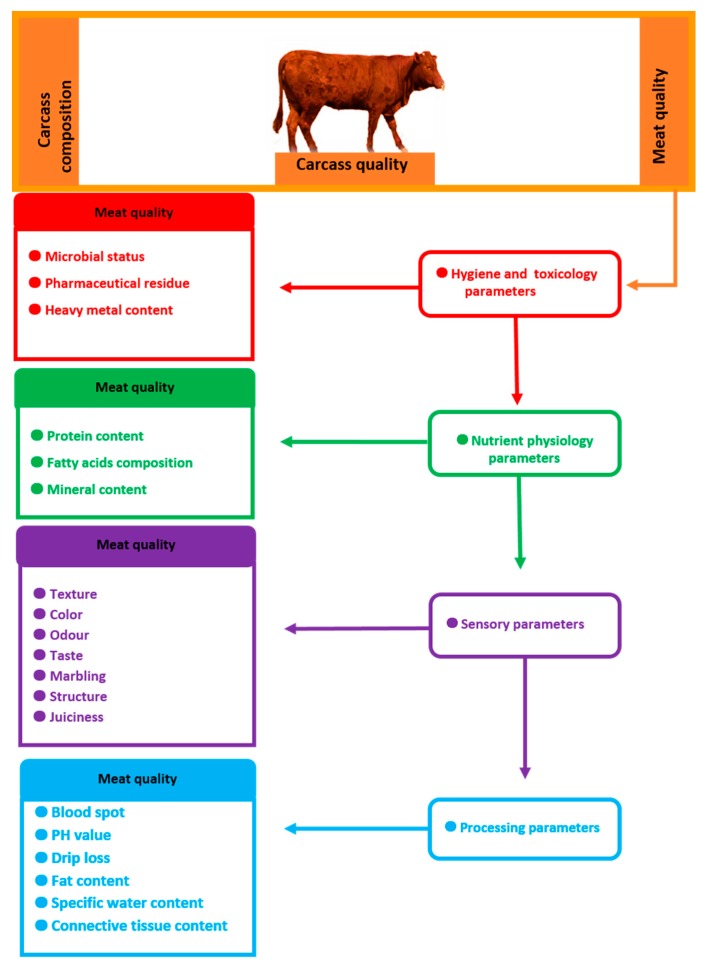
The most important carcass and meat quality traits for meat-producing animals.

Intramuscular proteins of the connective tissue and myofibrillar proteins are the components that are considered key contributors to the tenderness of beef. Their action is largely determined by the muscle type, structure and composition, and also by the cooking method and temperature [27]. Another essential factor influencing the ultimate tenderness of beef is how the meat is stored and proteolytic activity. At a temperature above the freezing point, meat is subject to the processes of aging, which increases its tenderness and can influence taste. The changes that occur in meat as a result of endogenous proteolysis affect the structure and properties of both intramuscular connective tissue and muscle fibers. Connective tissue proteolysis during maturation of meat is reflected in increased collagen solubility, changes in the mechanical properties of perimysium, and changes in the composition of proteoglycans [26,28]. During maturation, myofibril structure undergoes breakdown of Z-line sarcomeres, some myofibril regulatory proteins, and thick and thin filaments of cytoskeletal proteins which stabilize the spatial arrangement. The proteolytic changes in these proteins occur under the influence of endogenous sacroplasmic proteinsthe calpains. The calpain system is represented by several isomeric cysteine proteases and their inhibitor calpastatin [9,29]. A great deal of studies has attempted to determine the function of genes whose polymorphism may impact the fattening and slaughter value of cattle as well as beef quality traits. However, the effect of the analyzed markers on different traits is not universal for all cattle breeds and cannot be extrapolated to the entire species [9,30,31]. The most important carcass and meat quality traits for meat-producing animals are summarized in Figure 2.

## 4. Gene Polymorphism vs. Slaughter Value and Beef Quality

It is difficult to improve meat quality traits based on conventional selection methods because they are regulated in a polygenic manner and have low heritability. The evaluation of meat quality characteristics is expensive and can only be carried out postmortem. Knowledge about the genes and chromosome regions associated with desired meat quality characteristics may prove very helpful when selecting for breeding and estimating the breeding value of offspring. Many studies have been carried out worldwide to establish the functions of various genes as well as polymorphisms with potential effects on the fattening and slaughter value of cattle, as well as on beef quality (Table 1). Table 2 also expresses all the included polymorphisms.

### 4.1. The SIRT4 Gene

The bovine *SIRT4* gene, which is expressed within the mitochondrial matrix and sited on chromosome 17, has three introns and nine exons. Functionally, it is linked with various multiple biological pathways; regulates lipid metabolism and, in particular, is associated with obesity-linked syndromes (i.e., cardiovascular and diabetes disease) in humans [77]; and maintain genomic stability [78]. *SIRT4* has a role in suppressing the oxidation of fatty acids and elevating lipogenesis by suppressing malonyl CoA decarboxylase activity [32]. The knockdown of *SIRT4* expression leads to an increase in peroxisome proliferator-activated receptor α (PPARα) expression in the liver, cellular respiration, and pAMPK levels and, subsequently, altered rates of fatty acid oxidation [33]. Elevation in the expression of the *SIRT4* gene may led to decreased adipocyte differentiation in the liver [34]. Recently, Gui et al. [11] detected polymorphisms in Qinchuan cattle *SIRT4* that are related with fat deposition and meat quality. They identified two SNPs in *SIRT4*; SNP (g.−311C > T) was shown to be associated to subcutaneous fat depth values, whereas the SNP (g.−1022G > A) had an effect on intramuscular fat content and subcutaneous fat depth values [11]. This was the first work to reference that *SIRT4* may directly or indirectly affect the quality traits of meat in Chinese cattle. Mammalian *SIRT4* gene expression is situated in the mitochondria, which regulates two biological functions related to mono-ADP ribosyltranferase and adeacetylase [79]. The *SIRT4* has resulted in suppressing fatty acid oxidation, eventually stimulating lipid anabolism in muscle cells via the repression of malonyl-CoA decarboxylase function [33]. Another mechanism of *SIRT4*, via its capability to catalyze deacetylation of malonyl-CoA decarboxylase, at the same time impedes fatty acid catabolism [35]. Under elevated ambient temperatures, *SIRT4* expression increases, affecting the metabolism of fatty acids in humans [80].

*SIRT4* is a central regulator of different metabolic pathways, particularly in the homeostasis of fats. In addition, *SIRT4* intermediates, either directly or indirectly affect the deposition of lipids in farm animals. Two SNPs markers (g.−311C > T and g.−1022C > A) of *SIRT4* could be used to identify bovine capable of accumulating lipids and meet carcass specifications. The *SIT4* polymorphisms are potential biomarkers for the selection of fat deposition and meat quality in livestock.

### 4.2. SIRT6 Gene

**Figure 3:**
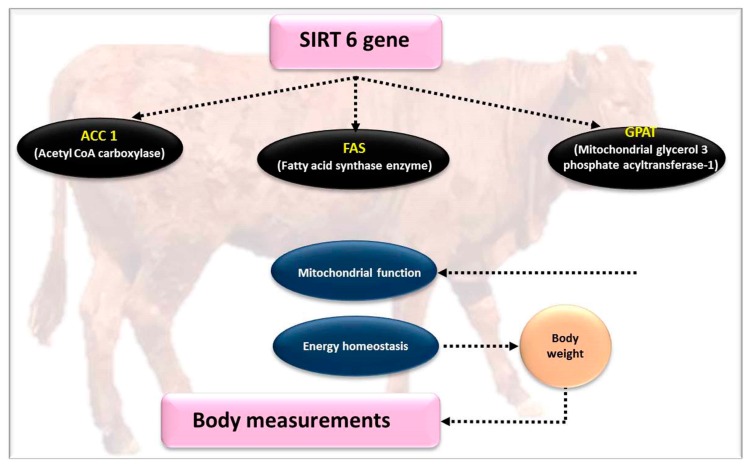
The effect of the *SIRT6* gene on meat quality parameters.

**Figure 4:**
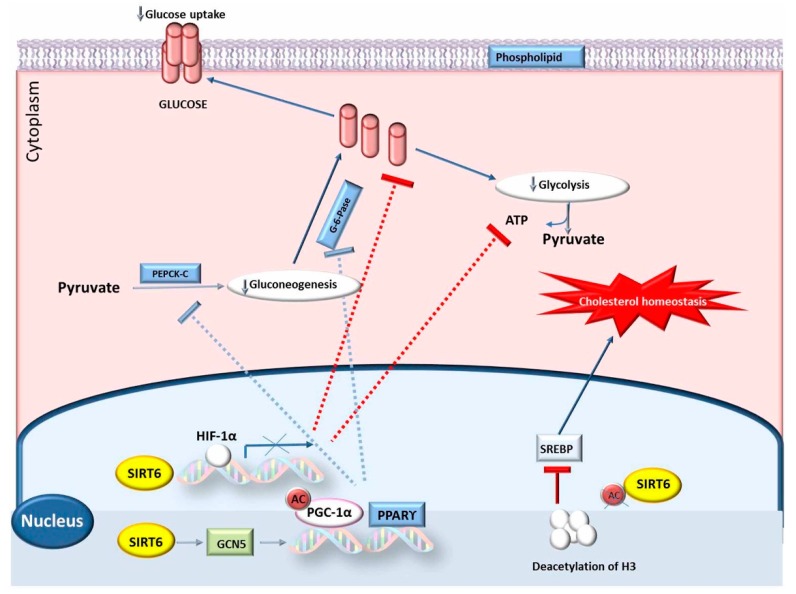
The mechanism of the *S1RT6* gene effect on growth parameters.

The mammalian sirtuins family of genehas seven homologs, *SIRT1* to *7*, are also considered as a part of the class III nicotinamide adenine dinucleotide-dependent deacetylase family [12]. The silent information regulators 6 (*SIRT6*) gene play a central role in carboxyl termini and other several cellular localizations in mammals [81] (Figure 3 and Figure 4). *SIRT6* is principally a helper protein with several biological pathways, such as nuclear chromatin, stress resistance, and lifespan, as well as has roles in metabolism [82]. Previous studies have indicated that *SIRT6* can deacetylate histone H3K9, and also modify the expression levels of genetic biomarkers linked with metabolism [36], and insufficient levels of this *SIRT6* gene expression in the liver may affect lipid and glycolysis metabolism [39,83]. Interestingly, the *SIRT6* gene attained was connected with cholesterol homeostasis of animals, and inhibits lipogenic transcription factors (i.e., *SREBP1* and *SREBP2*) through stimulating the mitochondrial phosphorylation process [37]. The *SIRT6* gene is necessary enzyme for metabolism of lipid, which is associated to fat deposition in mammals. Therefore, due to suppression of *PPARγ* gene, the transgenic mice expressed the *SIRT6* gene and downregulated the fat deposition in response to restricted feed intake by adjusting transcription factor binding sites, as previously described [84]. Gui et al. [38] have identified four sequence variants (SVs) in intron 6, exon 7, exon 9, and 3’UTR, using a technology based on sequencing that was carried out in 468 individual Qinchuan cattle. Four SNPs have been determined in the *SIRT6* gene of Qinchuan cattle, which are present in intron 6, exon 7, exon 9, and 3’UTR, respectively, using the same methods in 468 Qinchuan cattle [38,85].

The above-mentioned SNPs are closely related to body measurements and carcass quality traits in comparison with other genotypes [38,86]. Recently, the thickness of backfat and intramuscular fat was greater in individuals that inherited genotype -1100GG, than in individuals with the AA or GA genotype-1100AA), while genotypes of other SNPs (c.−84 C > T) in the promoter region of *SIRT6* had no significant association with deposition of lipids [39]. It seems that backfat thickness and intramuscular fat content are more represented in animals with a higher expression of the *SIRT6* gene (especially, SNP c.−1100 A > G) [2]. The role of *SIRT6* in regulating glucose and lipid metabolism is illustrated in Figure 3.

### 4.3. Silent Information Regulators 7 (SIRT7)

**Figure 5:**
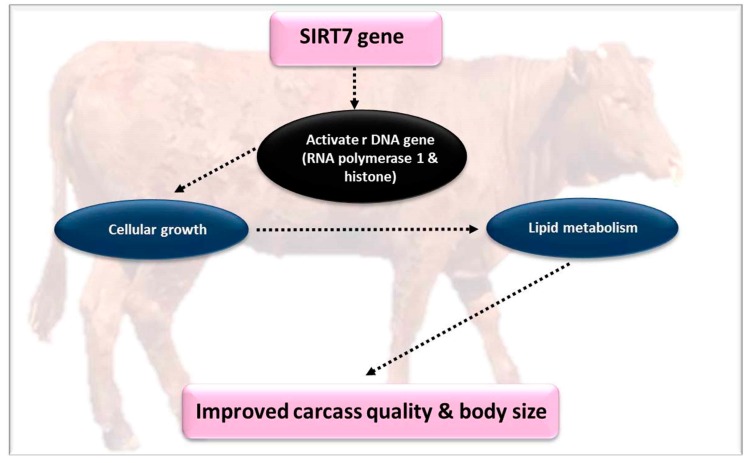
The effect of the *SIRT7* gene on meat quality parameters.

The *SIRT7* gene is the protein that is mostly correlated with rRNA activation genes, RNA polymerase 1 (RNA Pol 1), and histones [87], and has different roles in lipid and cellular homeostasis in sheep [40]. *SIRT7* is mostly localized in the nucleolus in mammals. In this way, it can regulate cell functions by acting as a cellular regulative protein with a mono-ribosyltransferase function. The expression of *SIRT7* upregulates RNA Pol 1-mediated rDNA transcription, while repression decreases the rDNA transcription. Likewise, *SIRT7* controls the differentiation of myoblasts and adipocytes, glucose homeostasis, cellular growth, as well as regulating lipid metabolism in the liver of mammals [40,41,42] (Figure 5).

The *SIRT7* gene controls glycolysis as well as lipid hemostasis [43,44] in the liver by regulating the ubiquitin-proteasome pathway [41,44,45]). Recent results demonstrated that *SIRT7* could modulate the TGF-β signaling pathway to restrain metastasis of tumors of the mammary gland [45], and also promote the activation of the ERK/STAT3 signaling pathway in glioma invasion and proliferation [88]. That showed that the *SIRT7* gene behaved as a key enzyme for lipid metabolism, which could be related to deposition of lipids in mammals. Previously, in Qinchuan cattle, two SNPs were identified in the *SIRT7* gene (exon 6 and exon 7): SNP2 (g.3587C > T) and SNP3 (g.3793T > C), which are linked with body traits and meat quality [15]. Furthermore, SNP2 (g.3587C > T) was determined in the *SIRT7* gene, and it was concluded that it can be linked with body size traits including a higher hip width and greater ultrasound loin muscle area in Qinchuan cattle [11,15]. Additionally, the same Chinese researchers found that the local cattle with an SNP3-C 3C3 genotype (g.3793T > C) had a significantly higher hip width, body length, ultrasound loin muscle area, chest circumference, and back fat thickness compared with those with the SNP3-T 3T3 genotype (*p* < 0.05).

### 4.4. CREB-Regulated Transcription Coactivator 3 (CRTC3)

CREB-regulated transcription co-activator 3 (CRTC3) is an essential protein in lipid homeostasis [89] and is regarded as a member of the CREB co-activator class (CREB-regulated transcription coactivators CRTC) [50]. *CRTC3* is placed on chromosome 21 in cattle and is contained in 15 exons and 14 introns. *CRTC* regulate the production of ATP via involvement of the c-AMP pathways [90,91,92,93] and encompass an N-terminal CREB binding area, a splicing domain, a central regulatory area, and a C-terminal transactivation region [46]. *CRTC* mRNA and protein are mainly present in white adipose tissue in humans. Expression analyses form previous reports suggested that the *CRTC3* gene is predominately expressed in both types of adipose tissues, and consequently stimulated by catecholamine activity [46]. It was also revealed that *CRTC3* controls lipid breakdown, mitochondrial production, and oxidation of fatty acids [47,48,49]. Decreased plasma free fatty acid levels were examined in the skeletal muscles of *CRTC3* knocked-out mice, while the results indicated reduced insulin sensitivity. In addition, the *CRTC3* gene promotes the capacity of mitochondrial oxidative process in muscle by overexpression of the hormone receptor coactivator *PGC1α* in the nucleus [46,48]. Besides, it was found that the polymorphisms of *CRTC3*, rs3862434 and rs8033595, are linked to obesity risk. Based on the function as a regulator in fat deposition, *CRTC3* is likely to influence traits related to fat deposition in animals.

**Figure 6:**
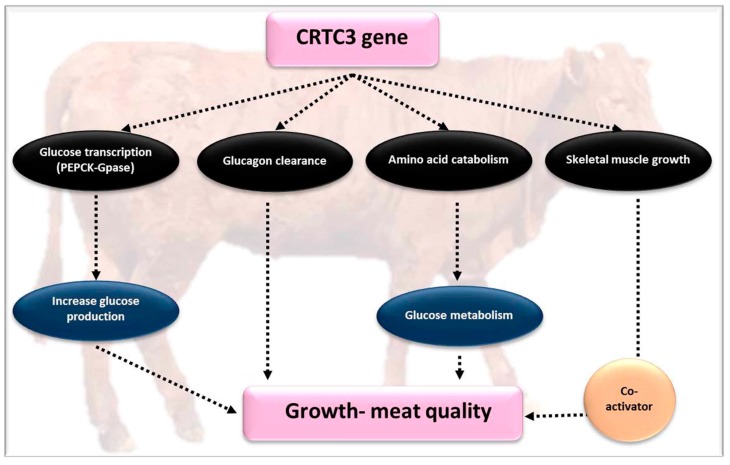
The role of *CRTC3* in regulating glucose and lipid metabolism.

For meat quality in native Chinese beef cattle, there were four SNPs, two in introns (SNP1: g.62652 A > G and SNP4: g.91297C > T) and two in exons (SNP2 g.62730C > T and SNP3: g.66478G > C) with an influence on carcass characteristics [50]. These SNPs primarily expressed in *CRTC3* which is located on chromosome 21. Animals with genotype AG, at the SNP1 locus, displayed a greater loin muscle area value than the other genotypes (*p* < 0.01). Unlike other genotypes (*p* < 0.05), at the SNP2 locus, with genotype CC, a greater BL, HH, RL, and HW was found. It can be concluded that the G allele could be used for trait selection in cattle at the SNP2 locus. Higher values of CD and BL were found in animals with genotype GC at the SNP3 locus, unlike the ones with genotype GG (*p* < 0.05 and *p* < 0.01). The cattle with genotype CT at SNP4 had a larger conformation (*p* < 0.05). The T allele in SNP4 is claimed to be related to better growth traits in indigenous Chinese cattle. Wu et al. [50] indicated high expression profiles in adipose tissues, rumen, and other splanchnic tissues. It is considered that *CRTC3* SNPs are linked to growth and carcass traits in bovine beef. Identification of genetic markers representing *CRTC3* SNPs is associated with traits of conformation and carcass, which can be used to advance marker-assisted selection and breeding programs in Chinese cattle for promoting the selection of economically favorable traits. The effect of the *CRTC3* gene on growth and meat quality parameters is summarized in Figure 6.

### 4.5. The α/β Hydrolase Domain-Containing Protein 5 (ABHD5)

**Figure 7:**
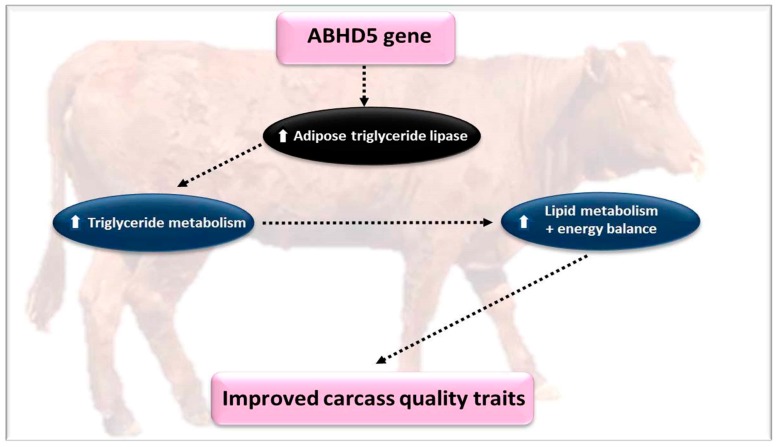
The effect of the *ABHD5* gene on growth and meat quality parameters.

The α/β-hydrolase fold domain containing protein 5 (ABHD5) is a part of the paralogous protein pair ABHD4 and ABHD5 that have a role as a co-activator of mammalian adipose triglyceride lipase (ATGL) and also participates in lipid metabolism and energy balance [16]. The control of intramuscular fat deposition is modulated by several lipolytic enzymes including: *ATGL*, *monoglyceride lipase* (*MAGL*), and hormone sensitive lipase (HSL). Consequently, it has been reported that the marbling trait of Hanwoo cattle was noticeably increased after castration, and protein and mRNA levels of *MAGL* and *ATGL* declined [51]. Castrated animals promote intramuscular fat accumulation via the reduction of lipolytic enzymes activity such *ATGL* and *MAGL*. Previous reports in different animals, for instance Wujin pigs with increased intramuscular fat deposition, had subordinate levels of *ATGL* expression than Shamrock pigs [52]. Enhanced triglyceride and fat accumulation were observed in the skeletal muscle of *ATGL* knockout mice, which exhibited reduced responsiveness to insulin [94]. Additionally, ABHD5 is one of the potential proteins which directly modifies ATGL and does not depend on the environment of the cell [53,95,96]. The *ABHD5* gene was identified as elevating the triglyceride hydrolase activity of ATGL the most [53]. ATGL lipase activity increases due to the presence of *ABHD5*, but also expands the substrate specificity. In the triglyceride, the acyl residue is primarily hydrolyzed by *ABHD5* at the sn-2 position. However, the presence of *ABHD5* supports the acyl residue at the sn-1 or sn-2 position [54]. Based on the fact that ABHD5 regulates ATGL function, it can be concluded that this protein has a role in the metabolism of lipids and energy balance [16]. The sequence analysis of 5′-regulated region of the *ABHD5* gene showed significant transcription factor binding sites such as CREB, C/EBP α, or PPAR γ, which indicates that the transcription of the *ABHD5* gene is tightly connected with the lipid metabolism and energy balance signaling pathways. Wang et al. [16] studied the regulation of cellular lipid metabolism in bovine adipocytes by the transcriptional regulatory mechanism of the *ABHD5* gene. The same researchers quantified the mRNA expression level of *ABHD5* in various tissues in differing generations of Qinchuan cattle and using molecular techniques, like gene cloning, the luciferase reporter assay, and site directed mutation and EMSA to identify the function of *Evi1* and *C/EBP*α transcription factors in the regulation of *ABHD5*. Wang et al. [16] indicated that the *ABHD5* gene was regulated by an ectopic viral integration site-1 (*Evi1*) and enhancer binding protein alpha (*C/EBP*α), and can be potential markers in marker-assisted selection, to develop a high-quality carcass in the mentioned breed. Such results show that *ABHD5* can be used as a genetic biomarker for marbled beef (Figure 7).

### 4.6. Kruppel-Like Factor 6 (KLF6)

**Figure 8:**
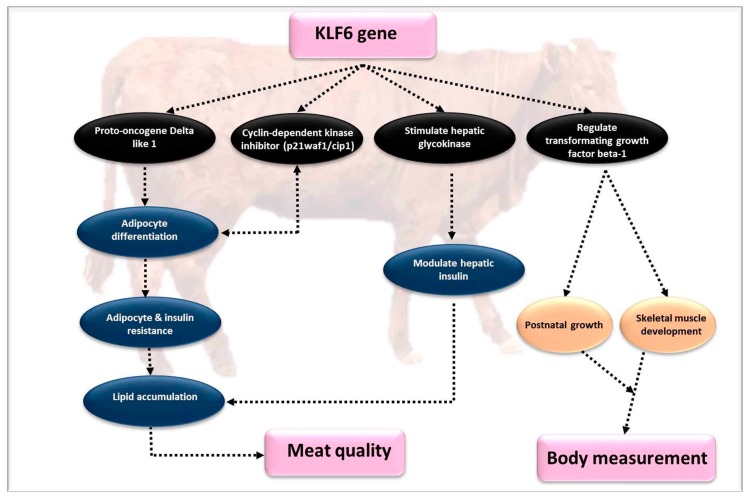
The effect of the *KLF6* gene on growth and meat quality parameters.

*KLF6* is a part of the mammalian sp1/KLF transcription factors, which regulate muscle development, cell division, development, differentiation, and adipogenesis [97,98,99,100]. *KLF6* is essential for adipogenesis [101]. Expression of *KLF6* mRNA or proteins has been documented in different tissues including liver, lungs, and kidney of yak [55], but the exact role and expression levels in cattle tissues has not been identified. The potential function of *KLF6* is as a transcriptional prohibitory factor of *Delta-like 1* (*Dlk1*) acting during the differentiation stages of adipocyte formation, The *KLF6* works by encoding a transmembrane protein that impedes adipogenesis [56]. The growth repressive activity of *KLF6* was observed in postnatal and developing skeletal muscle as controlling the cell cycle by transcriptional initiation of the cyclin-dependent kinase inhibitor p21WAF1/Cip1 [57]. By regulating the *transforming growth factor beta 1* (*TGFB1)*, the functions of *KLF6* in postnatal growth and development of skeletal muscles was determined [58,59], including the activation of hepatic glucokinase and regulation of insulin sensitivity in liver by NAFLD [102]. Recently, Raza et al. [3] evaluated the variations and haplotype combinations of the *KLF6* gene, and found three SNPs (3332C > G, 3413C > T, and 3521G > A) which are located in the 2nd exon of the bovine *KLF6* gene were associated with carcass quality and body measurements of Qinchuan cattle (Figure 8).

Expression of *KLF6* in the liver of bovines indicates that *KLF6* is involved in complex metabolism pathways and is a critical regulator of metabolic process and apoptosis [56,60]. The haplotype combination Hap1/4 of the *KLF6* gene was associated with a taller wither height, longer body length, wider hips, longer rump, more intramuscular fat, and a greater loin area compared to other haplotype combinations [3]. The Hap1/4 haplotype of *KLF6* is a potential biomarker for determination of body measures and carcass traits in Qinchuan cattle. Bearing in mind that the main functions of the *KLF6* gene are related to the lipid metabolism, there is a need for additional studies to identify the specific functions regarding meat quality traits, but also detailed polymorphism identification of other genes is necessary to clarify genotypes for body and carcass traits.

### 4.7. Heart Type Fatty Acid Binding Protein (H-FABP)

**Figure 9:**
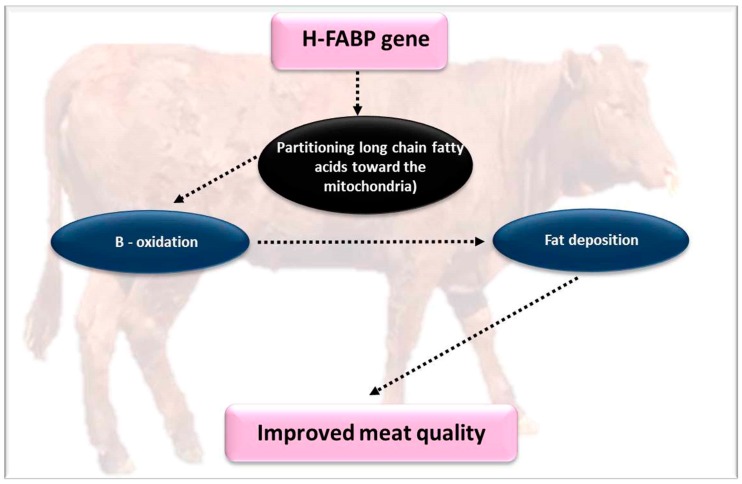
The effect of the *H-FABP* gene on growth and meat quality parameters.

Heart type fatty acid binding protein (H-FABP) is categorized as an intracellular fatty acid-binding protein used for the transportation of long-chain fatty acids. The *H-FABP* gene is expressed in the heart, subcutaneous fat, and skeletal muscles, and to a lesser extent expressed in brown fat tissue, placenta, and the neuron cells [103,104]. The *H-FABP* gene participates in signal transduction pathways, such as mitochondrial β oxidation and the uptake or utilization of long chain fatty acids [105]. *H-FABP* is associated with intramuscular fat concentration in pigs, which makes this biomarker an important candidate gene for intramuscular fat regulation [61,62] (Figure 9).

Previous studies reported that the H-FABP-null mice exhibited better insulin sensitivity, which was possibly associated to the augmented reliance on glucose [63]. The expressions of genes related to lipid metabolism and glycolysis are regulated by the knockdown of the *H-FABP* gene in brown adipocytes [106]. Li et al. [64] report that the *KLF15* gene can change the core promoter of the *H-FABP* gene, thus influencing the meat and growth traits in mammals. A SNP g.6643C > T in the promoter region of the Yak *H-FABP* gene is linked to weight and body length via modification of several transcription factors binding sites and through diverting long-chain fatty acids to the mitochondria [11,65].

The *H-FABPs* are related to IMF and a low (lean meat) fat percentage [66,81]. Higher expression levels of *H-FABP* stimulate adipogenesis in preadipocytes [67]. Even though it seems that this gene does not have any significant functions in determining quality traits, like carcass weight and backfat [107], previous studies on the native Tibetan pig stated that the levels of mRNA and protein expression of *H-FABP* in backfat, longissimus dorsi, and liver were increased in pigs with greater fat [62]. Shang et al. [62] reported that the C-1375G site can stimulate expression of *H-FABP* and in this way be connected to lipid deposition in pigs.

### 4.8. Very Long Chain Fatty Acids Protein 5 (ELOVL5)

**Figure 10:**
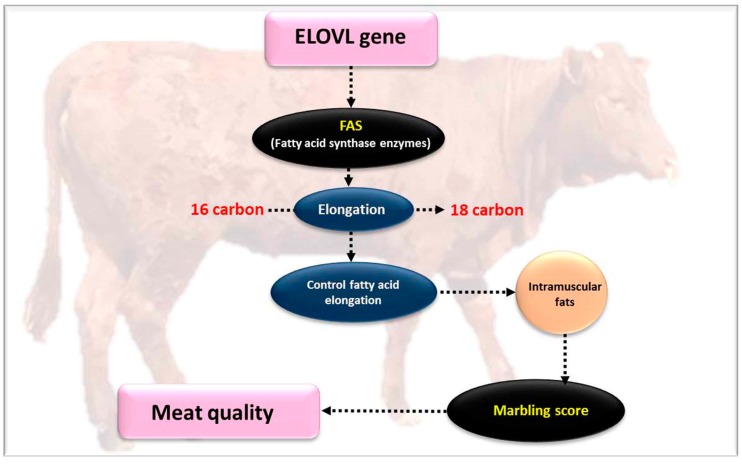
The effect of the *ELOVL5* gene on meat quality parameters.

ELOVL and its homologs are parts of the very long chain fatty acids protein class (Figure 10). Seven homologues of ELOVL have been identified (ELOVL 1–7) and are identified as being involved in fat metabolism in mammals. The *ELOVL* genes encode enzymes which are differentially represented in different tissues, and the individual enzymes have different preferences of fatty acid substrate [108,109]. Among them, *ELOVL5* is involved in the synthesis of palmitic acid (C16:0), palmitoleic acid (C16:1), stearic acid (C18:0), and oleic acid (C18:1) [70], which are the main fatty acids in beef [2,68]. ELOVL5 is an essential protein in for the synthesis of specific monounsaturated fatty acid (MUFA) in mammalian cells and has a role in fatty acid elongation [70]. When *ELOVL5* is knocked out, the elongation of C16:1 cis-9, n-7 is reduced, while the overexpression of *ELOVL5* induces an enhanced fatty acid synthesis of C18:1 cis-9, n-7 [68]. Furthermore, it has been confirmed that the reduction of *ELOVL5* activity is associated with increased risk for hepatic steatosis, while polyunsaturated fatty acids (PUFAs) that are internal produced are key regulators of fatty acid synthesis [69].

Studies in pigs have proven that the *ELOVL5* gene takes part in fatty acid production and is associated with C20:1n9/C18:1n9 and C20:2n6/C18:2n6 production [71]. *ELOVL5* can have pleiotropic effects on diverse fatty acid structures in multiple stages of the metabolism. Recently, Zhu et al. [72] performed genome-wide association study (GWAS)in Chinese Simmental cattle and they concluded that the *ELOVL5* gene was associated with C14:0 in Chinese Simmental cattle. It could be seen that different regions and loci can be candidate biomarkers for genomics-based breeding strategies. All the mentioned methods have proved that *ELOVL5* is strongly linked with fatty acids, and can be used for genomic selection for fatty acids in the Chinese Simmental cattle.

### 4.9. Very Long Chain Fatty Acids Protein 6 (ELOVL6)

Very long chain fatty acids protein 6 (*ELOVL6*) gene encodes a crucial protein that participates in lipogenesis by catalyzing the elongation of monounsaturated and saturated fatty acids [17]. Expression of bovine *ELOVL6* has been confirmed in many tissues important in lipid metabolism, including adipose tissues, brain and the liver. The *ELOVL6* gene takes part in the biosynthesis of several fatty acids, including; palmitic acid (C16:0), palmitoleic acid (C16:1), stearic acid (C18:0) and oleic acid (C18:1). Additionally, *ELOVL6* controls the elongation of C16:0 (palmitate) to C18:0 (stearate). Because palmitate and stearate are the major fatty acids in beef [68], the participation of ELOVL6 in the production of fatty acids may be especially important for beef breeding.

The deficiency expression of *ELOVL6* in mice has improved quantities of C16:0 and C16:1n-7 in various tissues, and reduced levels of C18:0 and C18:1n-9 [108,110,111]. Recent studies suggest, that the stearic acid (C18:0), as an *ELOVL6* product, participates in the control of mitochondrial function via stearoylation of *transferring receptor* 1 (*TFR1*) [112]. *ELOVL6* lowers the levels of mRNA expression in genes that are in charge for esterification, such as concentrations of glycerol-3-phosphate acyltransferase mitochondrial and *diacylglycerol acyltransferase 2* (*DGAT2*), and also triacylglycerol [73]. Recent studies proved that *ELOVL6* is a direct target of sterol regulatory element-binding protein 1 (SREBP-1) and is regulated primarily by SREBP-1c and carbohydrate-responsive element-binding protein (ChREBP) [74,75,76]. Binding of *SP1* to the promoter regions of *ELOVL6* controls their transcription activity [113]. The *ELOVL6* c.-394G > A polymorphism is responsible for mutation of the quantitative trait locus on pig chromosome 8, what in the end dictates porcine fatty acid composition [61]. Results of transcriptome and genomic sequencing reveal that *ELOVL6* is a potential biomarker that affects meat quality in bovines and has an important role in the regulation of fat metabolism by elongating fatty acids [17].

Junjvlieke et al. [17] reported that the binding of transcription factors *KLF6* and *PU.1* appeared in the −168/+69 region, and have an essential role in regulating the transcription of bovine *ELOVL6*. The same researchers claim that up-regulation of *ELOVL6* increases *peroxisome proliferator activated receptor γ* (*PPARγ*) expression, however it down regulates the fatty acid-binding protein 4 (FABP4) expression. Additionally, the reduction of *ELOVL6* subsequently regulated the expression level of mRNA *PPARγ*, *FABP4*, *ACSL*, and *FATP1*. By regulating the expression levels of genes involved in the cell cycle, *ELOVL6* stimulates adipogenesis.

## 5. Conclusions

Marker-assisted selection is a powerful tool for the improvement of beef cattle production, and can be used to advance both management and breeding decisions. In this review, we summarized numerous regions and SNPs that are potential biomarkers for genomics-assisted breeding strategies for the improvement of carcass quality and body variables in Chinese Qinchuan cattle. It appears that there is a need for further studies to assess the potential role of other sequences of the major genes or other associated genes in different breeds and populations. Novel methods, like whole-genome sequencing and whole-transcriptome profiling, are likely to be valuable tools to identify signatures of selection and gene pathways involved in these traits.

## Figures and Tables

**Table 1:** Gene association with meat quality in cattle.

Gene Type	Author(s)	Findings
The *SIRT4* gene	[32]	Stimulated in the purpose of suppressing the oxidation of fatty acid in obesity, further to elevate lipogenesis levels.
	[33]	Leads to an increase in peroxisome proliferator-activated receptor α (PPARα) expression in the liver, cellular respiration, and pAMPK levels and, subsequently, changed rates of fatty acid oxidation. Suppresses fatty acid oxidation, eventually stimulating lipid anabolism in muscle cells.
	[34]	Decreased adipocyte differentiation in the liver.
	[11]	They identified two SNPs in the *SIRT4* gene related to meat quality in Qinchuan cattle; SNP (g.-311C > T) was shown to be associated to subcutaneous fat depth values.
	[35]	Has capability to catalyze deacetylation of malonyl-CoA decarboxylase and at the same time impedes fatty acid catabolism.
*SIRT6* gene	[36]	Can deacetylate histone H3K9, also modify the expression levels of genetic biomarkers linked with metabolism.
	[37]	Connected with cholesterol homeostasis of animals, and inhibits lipogenic transcription factors (i.e., SREBP1 and SREBP2).
	[38]	Four SNPs have been determined in the *SIRT6* gene of Qinchuan cattle, which are present in intron 6, exon 7, exon 9, and 3’UTR, respectively, using the same methods in 468 Qinchuan cattle. The above-mentioned SNPs are closely related to body measurements and carcass quality traits in comparison with other genotypes.
	[39]	The thickness of back fat and intramuscular fat value of individuals that inherited genotype -1100GG, were found to be notably higher than in individuals with the AA or GA genotype -1100AA (*p* < 0.05).
	[2]	Back fat thickness and intramuscular fat content are more represented in animals with a higher expression of the *SIRT6* gene.
Silent information regulators 7 (*SIRT7*)	[40,41,42]	Controls the differentiation of myoblasts and adipocytes; glucose homeostasis, cellular growth, also regulating lipid metabolism in the liver of mammals.
	[43,44]	Controls the glycolysis as well as lipid hemostasis.
	[45]	Modulate the TGF- β signaling pathway to restrain metastasis of tumors of the mammary gland.
	[57]	Promote the activation of ERK/STAT3 signaling pathway in glioma invasion and proliferation.
	[15]	In Qinchuan cattle, two SNPs were identified in the *SIRT7* gene (exon 6 and exon 7): SNP2 (g.3587C > T) and SNP3 (g.3793T > C). SNP2 (g.3587C > T) was determined in the SIRT7 gene, and it was concluded that it can be linked with certain body size traits in Qinchuan cattle Qinchuan cattle with SNP3-C 3C3 genotype (g.3793T > C), had significantly higher hip width, body length, ultrasound loin muscle area, chest circumference, and back fat thickness.
CREB-regulated transcription coactivator 3 (*CRTC3*)	[46]	*CRTC3* gene is predominately expressed in both types of adipose tissues, and consequently stimulated by catecholamine activity.
	[47,48,49]	*CRTC3* controls lipid breakdown, mitochondrial production and oxidation of fatty acids.
	[46,48]	*CRTC3* gene promotes the capacity of mitochondrial oxidative process in muscle.
	[50]	*CRTC3* SNPs are linked to body masses and carcass traits in bovine beef. So, identification of genetic markers represented in *CRTC3* SNPs has a close association with traits of conformation and the carcass.
The α/β hydrolase domain containing 5 (*ABHD5*)	[51]	The marbling trait of Hanwoo cattle was noticeably increased after emasculation, while protein and mRNA levels of MAGL and ATGL have been declined.
	[52]	Wujin pigs with increased intramuscular fat deposition had subordinate levels of ATGL expression than Shamrock pigs.
	[53]	*ABHD5* gene was identified as elevating the triglyceride hydrolase activity of ATGL the most.
	[54]	ATGL lipase activity increases due to the presence of *ABHD5*, but also expands the substrate specificity.
	[16]	Indicated that the *ABHD5* gene regulated by Ectopic viral integration site-1 (Evi1) and enhancer binding protein alpha (C/EBP α), can be potential markers in MAS, to develop high-quality carcass in the mentioned breed. Such results show that *ABHD5* can be used as a genetic biomarker for marbled beef selection, which will be very useful in MAS for carcass quality.
Kruppel-like factor 6 (*KLF6*)	[55]	Expression analysis of *KLF6* mRNA or proteins has been documented in different tissues including liver, lungs, and kidney of yak.
	[56]	Potential function of *KLF6* during the differentiation of adipocyte it is stated to be a transcriptional prohibitory factor of Delta-like 1 (Dlk1).
	[57]	The growth repressive activity arbitrated via KLF6 protein that controls the cell cycle by transcriptional initiation of the cyclin-dependent kinase inhibitor p21WAF1/Cip1.
	[58,59]	By regulating the *TGFB1* signaling, the following functions of the *KLF6* gene were determined: Postnatal growth and development of skeletal muscles.
	[3]	Three SNPs (3332C > G; 3413C > T and 3521G > A) are located in the 2nd exon of the bovine *KLF6* gene, which consequently confirms their influence on carcass quality and body measurement in Qinchuan cattle. Hap1/4 is related in a greater amount to ULA and IF than other combinations
	[60]	The haplotype combination Hap1/4 was significantly related with withers height, greater body length, hip width, rump length, intramuscular fat, and ultrasound loin area.
	[56,60]	Overexpression of *KLF6* in the liver of bovine shows that *KLF6* has a remarkable and complex process in metabolism pathways.
Heart type fatty acid binding protein *(H-FABP)*	[61,62]	*H-FABP* is related with the pig intramuscular fat, which makes this biomarker an important candidate gene for intramuscular fat regulation.
	[63]	H-FABP-null mice exhibited better insulin sensitivity.
	[64]	*KLF15* gene can change the core promoter of the *H-FABP* gene.
	[11,65]	An SNP g.6643C > T in the promoter region of the bovine *H-FABP* gene is mostly linked to weight and body length (*p* < 0.01) in Yak via modification of several transcription factors binding sites.
	[66]	The analysis of single-markers showed that *H-FABPs* are related to IMF and a low fat percentage.
	[67]	Higher expression levels of *H-FABP* stimulate adipogenesis in 3T3-L1 preadipocytes.
Very long chain fatty acids protein 5 (*ELOVL5*)	[2,68]	*ELOVL5* is complicated in the synthesis of palmitic acid (C16:0), palmitoleic acid (C16:1), stearic acid (C18:0), and oleic acid (C18:1).
	[68]	The overexpression of *ELOVL5* induces an enhanced fatty acid synthesis C18:1 cis-9, n-7
	[69]	The reduction of *ELOVL5* activity is associated with increased risk for hepatic steatosis.
	[70]	*ELOVL5* has a role in the synthesis of various bovine acids, like C16:0, C16:1, C18:0, and C18:1.
	[71]	The *ELOVL5* gene took part in fatty acid production. *ELOVL5* was also proven to be related with C20:1n9/C18:1n9 and C20:2n6/C18:2n6 production.
	[72]	Concluded that the *ELOVL5* gene was associated with C14:0 in Chinese Simmental cattle.
	[70]	*ELOVL5* has a role in the synthesis of various acids in bovine, like C16:0, C16:1, C18:0, and C18:1.
Very long chain fatty acids protein 6 (*ELOVL6*)	[68]	*ELOVL6* controls the elongation of C16:0 (Palmitate) to C18:0 (Stearate) in beef.
	[73]	*ELOVL6* lowers the levels of mRNA expression in genes that are in charge for esterification.
	[74,75,76]	*ELOVL6* is a direct target of sterol regulatory element-binding protein 1 (SREBP-1), and is regulated primarily by SREBP-1c and the carbohydrate-responsive element-binding protein (ChREBP).

**Table 2:** Effects of various polymorphisms/variants as marker-assisted selection on body variables and meat quality traits in Qinchuan cattle.

Gene	SNP	Site	Related Traits	References
*SIRT4*	g.−311C > T and g.−771C	ND	Subcutaneous fat depths	[11]
	g.−1022G > A	ND	Intramuscular fat content subcutaneous fat depth	[11]
*SIRT6*	g.8460GNA	ND	Body measurements	[38]
	g.9429CNT	ND	Meat traits	[38]
	g.9735TNC	ND	Body measurements	[38]
*SIRT7*	g.3587C > T	Exon 6	Body size and meat quality traits	[11,15]
	g.3793T > C	Exon 7	Body size and meat quality traits	[11,15]
*CREC3*	g.62652 A > G	Intron 3	Loin muscle area	[50]
	g.62730C > T	Exon 4	BL, HH, RL, and HW	[50]
	g.66478G > C	Exon 6	BL and CD	[50]
	g.91297C > T	Intron 13	Body conformation	[50]
*ABHD5*	Evi1 and C/EBP α as a transcriptional factor	Transcriptional factor	Carcass quality traits	[16]
*KLF6*	g.3332C > G	Exon2	Body and carcass measurements	[3]
	g.3413C > T	Exon2	Body and carcass measurements	[3]
	g.3521G > A	Exon2	Body and carcass measurements	[3]
*H-FABP*	g.6643C > T	ND	Weight and body length	[11,65]
	g.1375 C >G	ND	Lipid deposition	[62]
*ELOVL5*	g.−110T>C	ND	Monounsaturated fatty acid, SFA saturated fatty acid	[2,68,70]
		ND	Subcutaneous fat thickness	[70]
			Fatty acid profile	
*ELOVL6*	ND	ND	Fatty acid profile in meat	[61]

Note: None detected (ND), body length (BL), chest depth (CD), hip height (HH), rump length (RL), and hip width (HW).

## References

[1] World Health Organization Q&A on the Carcinogenicity of the Consumption of Red Meat and Processed Meat. https://www.who.int/features/qa/cancer-red-meat/en/.

[2] Abd El-Hack M.E., Abdelnour S.A., Swelum A.A., Arif M. (2018). The application of gene marker-assisted selection and proteomics for the best meat quality criteria and body measurements in Qinchuan cattle breed. Mol. Boil. Rep..

[3] Raza S.H.A., Khan R., Schreurs N.M., Guo H., Gui L.S., Mei C., Zan L. (2019). Expression of the bovine KLF6 gene polymorphisms and their association with carcass and body measures in Qinchuan cattle (*Bos Taurus*). Genomic.

[4] Pedersen L.D., Sørensen A.C., Berg P. (2009). Marker-assisted selection can reduce true as well as pedigree-estimated inbreeding. J. Dairy Sci..

[5] Raza S.H.A., Gui L., Khan R., Schreurs N.M., Wang X., Wu C., Mei L., Wang X., Ma D., Wei H. (2018). Association between FASN gene polymorphisms ultrasound carcass traits and intramuscular fat in Qinchuan cattle. Gene.

[6] Boucher C.A., Carey N., Edwards Y.H., Siciliano M.J., Johnson K.J. (1996). Cloning of the human SIX1 gene and its assignment to chromosome 14. Genomics.

[7] D’Andre Hirwa C., Wallace P., Shen X., Nie Q., Yang G., Zhang X. (2011). Genes related to economically important traits in beef cattle. Asian J. Anim. Sci..

[8] Boukha A., Bonfatti V., Cecchinato A., Albera A., Gallo L., Carnier P., Bittante G. (2011). Genetic parameters of carcass and meat quality traits of double muscled Piemontese cattle. Meat Sci..

[9] Gao Y.U., Zhang R., Hu X., Li N. (2007). Application of genomic technologies to the improvement of meat quality of farm animals. Meat Sci..

[10] Ribeca C., Bonfatti V., Cecchinato A., Albera A., Gallo L., Carnier P. (2014). Effect of polymorphisms in candidate genes on carcass and meat quality traits in double muscled Piemontese cattle. Meat Sci..

[11] Gui L., Wu H., Raza S.H.A., Schreurs N.M., Shah M.A. (2019). The effect of haplotypes in the promoter region of SIRT4 gene on the ultrasound traits in Qinchuan cattle. Trop. Anim. Health Prod..

[12] Luft F.C. (2014). Are you certain about SIRT?. J. Mol. Med..

[13] Silberman D.M., Ross K., Sande P.H., Kubota S., Ramaswamy S., Apte R.S., Mostoslavsky R. (2014). SIRT6 is required for normal retinal function. PLoS ONE.

[14] Li X., Kazgan N. (2011). Mammalian sirtuins and energy metabolism. Int. J. Biol. Sci..

[15] Gui L., Xin X., Wang J., Hong J., Zan L. (2016). Expression analysis, single nucleotide polymorphisms within SIRT4 and SIRT7 genes and their association with body size and meat quality traits in Qinchuan cattle. J. Integr. Agric..

[16] Wang X., Khana R., Razaa S.H.A., Lia A., Zhanga Y., Lianga C., Yanga W., Wua S., Zan L. (2019). Molecular characterization of ABHD5 gene promoter in intramuscular preadipocytes of Qinchuan cattle: Roles of Evi1 and C/EBP α. Gene.

[17] Junjvlieke Z., Mei C.G., Khan R., Zhang W.Z., Hong J.Y., Wang L., Zan L.S. (2019). Transcriptional regulation of bovine elongation of very long chain fatty acids protein 6 in lipid metabolism and adipocyte proliferation. J. Cell Biochem..

[18] Mach N., Bach A., Velarde A., Devant M. (2008). Association between animal, transportation, slaughterhouse practices, and meat pH in beef. Meat Sci..

[19] Węglarz A. (2011). Effect of pre-slaughter housing of different cattle categories on beef quality. Anim. Sci. Pap. Rep..

[20] Chambaz A., Scheeder M.R.L., Kreuzer M., Dufer P.A. (2003). Meat quality of Angus, Simmental, Charolais and Limousin steers compared at the same level of intramuscular fat. Meat Sci..

[21] Keane M.G., Dunne P.G., Kenny D.A., Berry D.P. (2011). Effect of genetic merit for carcass weight, breed type and slaughter weight on performance and carcass traits of beef x diary steers. Animal.

[22] Magolski J.D., Buchanan D.S., Maddock-Carlin K.R., Anderson V.L., Newman D.J., Berg E.P. (2013). Relationship between commercially available DNA analysis and phenotypic observations on beef quality and tenderness. Meat Sci..

[23] Harper G.S. (1999). Trends in skeletal muscle biology and underestanding of toughness in beef. Aust. J. Agric. Res..

[24] Juszczuk-Kubiak E., Rosochacki S.J., Wicińska K., Słoniewski K., Łyczyński A., Sakowski T. (2007). Association of the SNP in the 3’-UTR region of the small subunit of bovine calcium-activated neutral protease (CAPN1S) with the activity of the calpain II and meat quality traits of Fresian bulls. Eurobiotech I Międzynarodowa Konferencja oraz Targi. Biotechnologia w Rolnictwie.

[25] Resurreccion A.V.A. (2004). Sensory aspects of consumer choices for meat and meat products. Meat Sci..

[26] Purslow P.P. (2005). Intramuscular connective tissue and its role in meat quality. Meat Sci..

[27] Brooks J.C., Savell J. (2004). Perimysium thickness as an indicator of beef tenderness. Meat Sci..

[28] Kołczak T., Pałka K., Pośpiech E. (2003). Changes in collagen solubility of raw and roasted bovine psoas major and minor and semitendinosus muscles during cold storage. Pol. J. Food Nutr. Sci..

[29] Maddock K.R., Huff-Lonergan E., Rowe L.J., Lonergan S.M. (2005). Effect of pH and ionic strength on μ-and m-calpain inhibition by calpastatin. J. Anim. Sci..

[30] Allais S., Journaux L., Levéziel H., Payet-Duprat N., Raynaud P., Hocquette J.F., Lepetit J., Rousset S., Denoyelle C., Bernard-Capel C. (2011). Effects of polymorphisms in the calpastatin and μ-calpain genes on meat tenderness in 3 French beef breeds. J. Anim. Sci..

[31] Reardon W., Mullen A.M., Sweeney T., Hamill R.M. (2010). Association of polymorphisms in candidate genes with colour, water-holding capacity, and composition traits in bovine m. longissimus and m. semimembranosus. Meat Sci..

[32] Laurent G., German N.J., Saha A.K., de Boer V.C.J., Davies M., Koves T.R., Dephoure N., Fischer F., Boanca G., Vaitheesvaran B. (2013). SIRT4 coordinates the balance between lipid synthesis and catabolism by repressing malonyl CoA decarboxylase. Mol. Cell.

[33] Nasrin N., Wu X., Fortier E., Feng Y., Bare O.C., Chen S., Ren X., Wu Z., Streeper R.S., Bordone L. (2010). SIRT 4 regulates fatty acid oxidation and mitochondrial gene expression in liver and muscle cells. J. Biol. Chem..

[34] Laurent G., de Boer V.C.J., Finley L.W.S., Sweeney M., Lu H., Schug T.T., Cen Y., Jeong S.M., Li X., Sauve A.A. (2013). SIRT4 represses PPARα activity to suppress hepatic fat oxidation. Mol. Cell Biol..

[35] Braidy N., Poljak A., Grant R., Jayasena T., Mansour H., Chan-Ling T., Smythe G., Sachdev P., Guillemin G.J. (2015). Differential expression of sirtuins in the aging rat brain. Front. Cell Neurosci..

[36] Michishita E., McCord R.A., Berber E., Kioi M., Padilla-Nash H., Damian M., Chang H.Y. (2008). SIRT6 is a histone H3 lysine 9 deacetylase that modulates telomeric chromatin. Nature.

[37] Elhanati S., Kanfi Y., Varvak A., Roichman A., Carmel-Gross I., Barth S., Cohen H.Y. (2013). Multiple regulatory layers of SREBP1/2 by SIRT6. Cell Rep..

[38] Gui L., Jiang B., Zhang Y., Zan L. (2015). Sequence variants in the bovine silent information regulator 6, their linkage and their associations with body measurements and carcass quality traits in Qinchuan cattle. Gene.

[39] Gui L.S., Raza S.H.A., Garcia M., Sun Y.G., Ullah I.R., Han Y.C. (2018). Genetic variants in the SIRT6 transcriptional regulatory region affect gene activity and carcass quality traits in indigenous Chinese beef cattle (*Bos taurus*). BMC Genom..

[40] Xu H., Zhang X., Zang R., Cai Y., Cao X., Yang J., Li J., Lan X., Wu J. (2019). Genetic variations in the sheep *SIRT7* gene and their correlation with body size traits. Arch. Anim. Breed.

[41] Yamagata K., Yoshizawa T. (2018). Transcriptional regulation of metabolism by SIRT1 and SIRT7. Int. Rev. Cell Mol. Biol..

[42] Shin J., He M., Liu Y., Paredes S., Villanova L., Brown K., Qiu X., Nabavi N., Mohrin M., Wojnoonski K. (2013). SIRT7 represses Myc activity to suppress ER stress and prevent fatty liver disease. Cell Rep..

[43] Jiang L., Xiong J., Zhan J., Yuan F., Tang M., Zhang C., Cao Z., Chen Y., Lu X., Li Y. (2017). Ubiquitin-specific peptidase 7 (USP7)-mediated deubiquitination of the histone deacetylase SIRT7 regulates gluconeogenesis. J. Biol. Chem..

[44] Ye X., Li M.T., Hou T.Y., Gao T., Zhu W.G., Yang Y. (2017). Sirtuins in glucose and lipid metabolism. Oncotarget.

[45] Tang B.L. (2015). SIRT7 and hepatic lipid metabolism. Front. Cell. Dev. Biol..

[46] Altarejos J.Y., Montminy M. (2011). CREB and the CRTC co-activators: Sensors for hormonal and metabolic signals. Nat. Rev. Mol. Cell Biol..

[47] Bachman E.S., Dhillon H., Zhang C.Y., Cinti S., Bianco A.C., Kobilka B.K., Lowell B.B. (2002). βAR signaling required for diet-induced thermogenesis and obesity resistance. Science.

[48] Wu Z., Huang X., Feng Y., Handschin C., Feng Y., Gullicksen P.S., Bare O., Labow M., Spiegelman B., Stevenson S.C. (2006). Transducer of regulated CREB-binding proteins (TORCs) induce PGC-1 α transcription and mitochondrial biogenesis in muscle cells. Proc. Natl. Acad. Sci. USA.

[49] Than T.A., Lou H., Ji C., Win S., Kaplowitz N. (2011). Role of cAMP-responsive elementbinding protein (CREB)-regulated transcription coactivator 3 (CRTC3) in the initiation of mitochondrial biogenesis and stress response in liver cells. J. Biol. Chem..

[50] Wu S., Yue N., Raza S.H.A., Chengtu Z., Le Z., Gong C., Hongbao W., Nicola S., Linsen Z. (2018). Genetic variants and haplotype combination in the bovine CRTC3 affected conformation traits in two Chinese native cattle breeds (*Bos Taurus*). Genomics.

[51] Bong J.J., Jeong J.Y., Rajasekar P., Cho Y.M., Kwon E.G., Kim H.C., Paek B.H., Baik M. (2012). Differential expression of genes associated with lipid metabolism in longissimus dorsi of Korean bulls and steers. Meat Sci..

[52] Zhao S.M., Ren L.J., Chen L., Zhang X., Cheng M.L., Li W.Z., Zhang Y.Y., Gao S.Z. (2009). Differential expression of lipid metabolism related genes in porcine muscle tissue leading to different intramuscular fat deposition. Lipids.

[53] Lass A., Zimmermann R., Haemmerle G., Riederer M., Schoiswohl G., Schweiger M., Kienesberger P., Strauss J.G., Gorkiewicz G., Zechner R. (2006). Adipose triglyceride lipase-mediated lipolysis of cellular fat stores is activated by CGI-58 and defective in Chanarin-Dorfman syndrome. Cell Metab..

[54] Eichmann T.O., Kumari M., Haas J.T., Farese R.V., Zimmermann R., Lass A., Zechner R. (2012). Studies on the substrate and stereo/regioselectivity of adipose triglyceride lipase, hormone-sensitive lipase, and diacylglycerol-O-acyltransferases. J. Biol. Chem..

[55] Goshu H., Wu X., Chu M., Bao P., Ding X., Yan P. (2018). Copy number variations of KLF6 modulate gene transcription and growth traits in Chinese Datong yak (*Bos Grunniens*). Animals.

[56] Li D., Yea S., Li S., Chen Z., Narla G., Banck M., Laborda J., Tan S., Friedman J.M., Friedman S.L. (2005). Krüppel-like factor-6 promotes preadipocyte differentiation through histone deacetylase 3-dependent repression of DLK1. J. Biol. Chem..

[57] Miele L., Beale G., Patman G., Nobili V., Leathart J., Grieco A., Abate M., Friedman S.L., Narla G., Bugianesi E. (2008). The Kruppel-like factor 6 genotype is associated with fibrosis in nonalcoholic fatty liver disease. Gastroenterol.

[58] Andreoli V., Gehrau R.C., Bocco J.L. (2010). Biology of Krüppel-like factor 6 transcriptional regulator in cell life and death. IUBMB Life.

[59] Dionyssiou M.G., Salma J., Bevzyuk M., Wales S., Zakharyan L., McDermott J.C. (2013). Krüppel-like factor 6 (KLF6) promotes cell proliferation in skeletal myoblasts in response to TGF β/Smad3 signaling. Skelet. Muscle.

[60] Ito G., Uchiyama M., Kondo M., Mori S., Usami N., Maeda O., Kawabe T., Hasegawa Y., Shimokata K., Sekido Y. (2004). Krüppel-like factor 6 is frequently downregulated and induces apoptosis in non-small cell lung cancer cells. Cancer Res..

[61] Zhang J., Cui L., Ma J., Chen C., Yang B., Huang L. (2017). Transcriptome analyses reveal genes and pathways associated with fatty acid composition traits in pigs. Anim. Genet..

[62] Shang P., Zhang B., Zhang J., Duan M., Wu L., Gong X., Tang K., Zhang H., Chamba Y. (2019). Expression and singlenucleotide polymorphisms of the H-FABP gene in pigs. Gene.

[63] Shearer J., Fueger P.T., Bracy D.P., Wasserman D.H., Rottman J.N. (2005). Partial gene deletion of heart-type fatty acid-binding protein limits the severity of dietary-induced insulin resistance. Diabetes.

[64] Li A., Wu L., Wang X., Xin Y., Zan L. (2016). Tissue expression analysis, cloning and characterization of the 5’-regulatory region of the bovine *FABP3* gene. Mol. Biol. Rep..

[65] Murphy E.J., Barcelo-Coblijn G., Binas B., Glatz J.F. (2004). Heart fatty acid uptake is decreased in heart fatty acid-binding protein gene-ablated mice. J. Biol. Chem..

[66] Chao Z., Wang F., Deng C.Y., Wei L.M., Sun R.P., Liu H.L., Liu Q.W., Zheng X.L. (2012). Distribution and linkage disequilibrium analysis of polymorphisms of MC4R, LEP, H-FABP genes in the different populations of pigs, associated with economic traits in DIV2 line. Mol. Biol. Rep..

[67] Yi B., Wang J., Wang S., Yuan D., Sun J., Li Z., Mao Y., Hou Q., Liu W. (2014). Overexpression of Banna mini-pig inbred line fatty acid binding protein 3 promotes adipogenesis in 3T3-L1 preadipocytes. Cell Biol. Int..

[68] Green C.D., Ozguden-Akkoc C.G., Wang Y., Jump D.B., Olson L.K. (2010). Role of fatty acid elongases in determination of de novo synthesized monounsaturated fatty acid species. J. Lipid. Res..

[69] Moon Y.A., Hammer R.E., Horton J.D. (2009). Deletion of ELOVL5 leads to fatty liver through activation of SREBP-1c in mice. J. Lipid Res..

[70] Matsumoto H., Shimizu Y., Tanaka A., Nogi T., Tabuchi I., Oyama K., Taniguchi M., Mannen H., Sasazaki S. (2013). The SNP in the promoter region of the bovine ELOVL5 gene influences economic traits including subcutaneous fat thickness. Mol. Biol. Rep..

[71] Zhang W., Bin Y., Zhang J., Cui L., Ma J., Chen C., Ai H., Xiao S., Ren J., Huang L. (2016). Genome-wide association studies for fatty acid metabolic traits in five divergent pig populations. Sci. Rep..

[72] Zhu B., Niu H., Zhang W., Wang Z., Liang Y., Guan L., Guo P., Chen Y., Zhang L., Guo Y. (2017). Genome wide association study and genomic prediction for fatty acid composition in Chinese Simmental beef cattle using high density SNP array. BMC Genom..

[73] Shi H.B., Wu M., Zhu J.J., Zhang C.H., Yao D.W., Luo J., Loor J.J. (2017). Fatty acid elongase 6 plays a role in the synthesis of long -chain fatty acids in goat mammary epithelial cells. J. Dairy Sci..

[74] Bae J.S., Oh A.R., Lee H.J., Ahn Y.H., Cha J.Y. (2016). Hepatic Elovl6 gene expression is regulated by the synergistic action of ChREBP and SREBP -1c. Biochem. Biophys. Res. Commun..

[75] Lee Y.J., Yu J.H., Kim W.H., Kim J.W. (2008). Mouse elovl-6 promoter is an SREBP target. Biochem. Biophys. Res. Commun..

[76] Matsuzaka T., Shimano H., Yahagi N., Yoshikawa T., Amemiya-Kudo M., Hasty A.H., Okazaki H., Tamura Y., Iizuka Y., Ohashi K. (2002). Cloning and characterization of a mammalian fatty acyl-CoA elongase as a lipogenic enzyme regulated by SREBPs. J. Lipid Res..

[77] Mariani S., Di Rocco G., Toietta G., Russo M.A., Petrangeli E., Salvatori L. (2017). Sirtuins 1–7 expression in human adipose derived stem cells from subcutaneous and visceral fat depots: Influence of obesity and hypoxia. Endocrine.

[78] Fernandez-Marcos P.J., Serrano M. (2013). Sirt4: The glutamine gatekeeper. Cancer Cell.

[79] Pannek M., Simic Z., Fuszard M., Meleshin M., Rotili D., Mai A., Schutkowski M., Steegborn C. (2017). Crystal structures of the mitochondrial deacylase Sirtuin 4 reveal isoform-specific acyl recognition and regulation features. Nature Commun..

[80] Acs Z., Bori Z., Takeda M., Osvath P., Berkes I., Taylor A.W., Yang H., Radak Z. (2014). High altitude exposure alters gene expression levels of DNA repair enzymes, and modulates fatty acid metabolism by SIRT4 induction in human skeletal muscle. Res. Physiol. Neurobiol..

[81] Han S.H. (2009). Potential role of sirtuin as a therapeutic target for neurodegenerative diseases. J. Clin. Neurol..

[82] Gan L., Mucke L. (2008). Paths of convergence: Sirtuins in aging and neurodegeneration. Neuron.

[83] Zhong L., D’Urso A., Toiber D., Sebastian C., Henry R.E., Vadysirisack D.D., Guimaraes A., Marinelli B., Wikstrom J.D., Nir T. (2010). The histone deacetylase Sirt6 regulates glucose homeostasis via Hif1 α. Cell.

[84] Pastinen T., Hudson T.J. (2004). Cis-acting regulatory variation in the human genome. Science.

[85] Chang M.T., Cheng Y.S., Huang M.C. (2012). A novel nonsynonymous SNP of the COLX gene and its association with duck reproductive traits. Mol. Cell. Probes.

[86] Guarente L. (2007). Sirtuins in aging and disease. Cold Spring Harbor Symposia Quantitat Biology.

[87] Ford E., Voit R., Liszt G., Magin C., Grummt I., Guarente L. (2006). Mammalian Sir2 homolog SIRT7 is an activator of RNA polymerase I transcription. Genes Dev..

[88] Mu P., Liu K., Lin Q., Yang W., Liu D., Lin Z., Shao W., Ji T. (2019). Sirtuin 7 promotes glioma proliferation and invasion through activation of the ERK/STAT3 signaling pathway. Oncol. Lett..

[89] Shan T., Xiong Y., Zhang P., Li Z., Jiang Q., Bi P., Yue F., Yang G., Wang Y., Liu X. (2016). Lkb1 controls brown adipose tissue growth and thermogenesis by regulating the intracellular localization of CRTC3. Nat. Commun..

[90] Khan R., Raza S.H.A., Junjvlieke Z., Xiaoyu W., Garcia M., Elnour I.E., Hongbao W., Linsen Z. (2019). Function and Transcriptional Regulation of Bovine TORC2 Gene in Adipocytes: Roles of C/EBP, XBP1, INSM1 and ZNF263. Int. J. Mol. Sci..

[91] Iourgenko V., Zhang W., Mickanin C., Daly I., Jiang C., Hexham J.M., Orth A.P., Miraglia L., Meltzer J., Garza D. (2003). Identification of a family of cAMP response element-binding protein coactivators by genome-scale functional analysis in mammalian cells. Proc. Natl. Acad. Sci. USA.

[92] Bittinger M.A., McWhinnie E., Meltzer J., Iourgenko V., Latario B., Liu L., Chen C.H., Song C., Garza D., Labow M. (2004). Activation of cAMP response element-mediated gene expression by regulated nuclear transport of TORC proteins. Curr. Biol..

[93] Screaton R.A., Conkright M.D., Katoh Y., Best J.L., Canettieri G., Jeffries S., Guzman E., Niessen S., Yates J.R., Takemori H. (2004). The CREB coactivator TORC functions as a calcium-and cAMP-sensitive coincidence detector. Cell.

[94] Haemmerle G., Lass A., Zimmermann R., Gorkiewicz G., Meyer C., Rozman J., Heldmaier G., Maier R., Theussl C., Eder S. (2006). Defective lipolysis and altered energy metabolism in mice lacking adipose triglyceride lipase. Science.

[95] Lu X., Yang X., Liu J. (2010). Differential control of ATGL-mediated lipid droplet degradation by CGI-58 and G0S2. Cell Cycle.

[96] Yang X., Lu X., Lombès M., Rha G.B., Chi Y.I., Guerin T.M., Smart E.J., Liu J. (2010). The G0/G1 switch gene 2 regulates adipose lipolysis through association with adipose triglyceride lipase. Cell Metab..

[97] Rowland B.D., Peeper D.S. (2006). KLF4, p21 and context-dependent opposing forces in cancer. Nat. Rev. Cancer.

[98] Kaczynski J., Cook T., Urrutia R. (2003). Sp1-and Krüppel-like transcription factors. Genome Biol..

[99] Ghaleb A.M., Katz J.P., Kaestner K.H., Du J.X., Yang V.W. (2007). Krüppel-like factor 4 exhibits antiapoptotic activity following γ-radiation-induced DNA damage. Oncogene.

[100] Suske G., Bruford E., Philipsen S. (2005). Mammalian SP/KLF transcription factors: Bring in the family. Genomics.

[101] Inuzuka H., Nanbu-Wakao R., Masuho Y., Muramatsu M., Tojo H., Wakao H. (1999). Differential regulation of immediate early gene expression in preadipocyte cells through multiple signaling pathways. Biochem. Biophys. Res. Commun..

[102] Bechmann L.P., Gastaldelli A., Vetter D., Patman G.L., Pascoe L., Hannivoort R.A., Lee U.E., Fiel I., Muñoz U., Ciociaro D. (2012). Glucokinase links Krüppel-like factor 6 to the regulation of hepatic insulin sensitivity in nonalcoholic fatty liver disease. Hepatology.

[103] Shioda N., Yamamoto Y., Watanabe M., Binas B., Owada Y., Fukunaga K. (2010). Heart-type fatty acid binding protein regulates dopamine D2 receptor function in mouse brain. J. Neurosci..

[104] Kusudo T., Kontani Y., Kataoka N., Ando F., Shimokata H., Yamashita H. (2011). Fatty acid-binding protein 3 stimulates glucose uptake by facilitating AS160 phosphorylation in mouse muscle cells. Genes Cells.

[105] Chen Q.M., Wang H., Zeng Y.Q., Chen W. (2013). Developmental changes and effect on intramuscular fat content of H-FABP and A-FABP mRNA expression in pigs. J. Appl. Genet..

[106] Vergnes L., Chin R., Young S.G., Reue K. (2011). Heart-type fatty acid-binding protein is essential for efficient brown adipose tissue fatty acid oxidation and cold tolerance. J. Biol. Chem..

[107] Lee S.H., Choi Y.M., Choe J.H., Kim J.M., Hong K.C., Park H.C., Kim B.C. (2010). Association between polymorphisms of the heart fatty acid binding protein gene and intramuscular fat content, fatty acid composition, and meat quality in Berkshire breed. Meat Sci..

[108] Jakobsson A., Westerberg R., Jacobsson A. (2006). Fatty acid elongases in mammals: Their regulation and roles in metabolism. Prog. Lipid Res..

[109] Tamura K., Makino A., Hullin-Matsuda F., Kobayashi T., Furihata M., Chung S., Ashida S., Miki T., Fujioka T., Shuin T. (2009). Novel lipogenic enzyme ELOVL7 is involved in prostate cancer growth through saturated long-chain fatty acid metabolism. Cancer Res..

[110] Matsuzaka T., Atsumi A., Matsumori R., Nie T., Shinozaki H., Suzuki-Kemuriyama N., Kuba M., Nakagawa Y., Ishii K., Shimada M. (2012). Elovl6 promotes nonalcoholic steatohepatitis. Hepatlogy.

[111] Matsuzaka T., Shimano H., Yahagi N., Kato T., Atsumi A., Yamamoto T., Inoue N., Ishikawa M., Okada S., Ishigaki N. (2007). Crucial role of a long -chain fatty acid elongase, Elovl6, in obesity-induced insulin resistance. Nat. Med..

[112] Senyilmaz D., Virtue S., Xu X., Tan C.Y., Griffin J.L., Miller A.K., Vidal-Puig A., Teleman A.A. (2015). Regulation of mitochondrial morphology and function by stearoylation of TFR1. Nature.

[113] Chen S., He H., Liu X.L. (2017). Tissue expression profiles and transcriptional regulation of elongase of very long chain fatty acid 6 in bovine mammary epithelial cells. PLoS ONE.

